# Quantifying metabolic activity of *Ascaris suum* L3 using resazurin reduction

**DOI:** 10.1186/s13071-023-05871-5

**Published:** 2023-07-19

**Authors:** Arkadi Kundik, Zaneta D. Musimbi, Jürgen Krücken, Thomas Hildebrandt, Oleg Kornilov, Susanne Hartmann, Friederike Ebner

**Affiliations:** 1 Institute of Immunology, Department of Veterinary Medicine, Freie Universität Berlin, Berlin, Germany; 2 Institute for Parasitology and Tropical Veterinary Medicine, Department of Veterinary Medicine, Freie Universität Berlin, Berlin, Germany; 3 Leibniz Institute for Zoo and Wildlife Research, Berlin, Germany; 4 Max-Born-Institute, Berlin, Germany; 5 Chair of Infection Pathogenesis, Department of Molecular Life Sciences, School of Life Sciences, Technical University Munich, Munich, Germany

**Keywords:** Drug screening assay, *Ascaris suum*, Larvae, Viability assay, Resazurin, Metabolic activity, Anthelmintics

## Abstract

**Background:**

Helminth infections are an important public health problem in humans and have an even greater impact on domestic animal and livestock welfare. Current readouts for anthelmintic drug screening assays are stage development, migration, or motility that can be subjective, laborious, and low in throughput. The aim of this study was to apply and optimize a fluorometric technique using resazurin for evaluating changes in the metabolic activity of *Ascaris suum* third-stage larvae (L3), a parasite of high economic relevance in swine.

**Methods:**

*Ascaris suum* L3 were mechanically hatched from 6- to 8-week embryonated and sucrose-gradient-enriched eggs. Resazurin dye and *A. suum* L3 were titrated in 96-well microtiter plates, and resazurin reduction activity was assessed by fluorometry after 24 h of incubation. Fluorescence microscopy was used to localize the resazurin reduction site within the larvae. Finally, we exposed *A. suum* L3 to various stress conditions including heat, methanol, and anthelmintics, and investigated their impact on larval metabolism through resazurin reduction activity.

**Results:**

We show that the non-fluorescent dye resazurin is reduced inside vital *A. suum* L3 to fluorescent resorufin and released into the culture media. Optimal assay parameters are 100–1000 L3 per well, a resazurin concentration of 7.5 µg/ml, and incubation at 37 °C/5% CO_2_ for 24 h. An intact L2 sheath around the L3 of *A. suum* completely prevents the uptake of resazurin, while in unsheathed L3, the most intense fluorescence signal is observed along the larval midgut. L3 exposed to methanol or heat show a gradually decreased resazurin reduction activity. In addition, 24 h exposure to ivermectin at 0.625 µM, mebendazole at 5 µM, and thiabendazole from 10 to 100 µM significantly decreased larval metabolic activity by 55%, 73%, and 70% to 89%, respectively.

**Conclusions:**

Together, our results show that both metabolic stressors and anthelmintic drugs significantly and reproducibly reduce the resazurin reduction activity of *A. suum* L3, making the proposed assay a sensitive and easy-to-use method to evaluate metabolic activity of *A. suum* L3 in vitro.

**Graphical Abstract:**

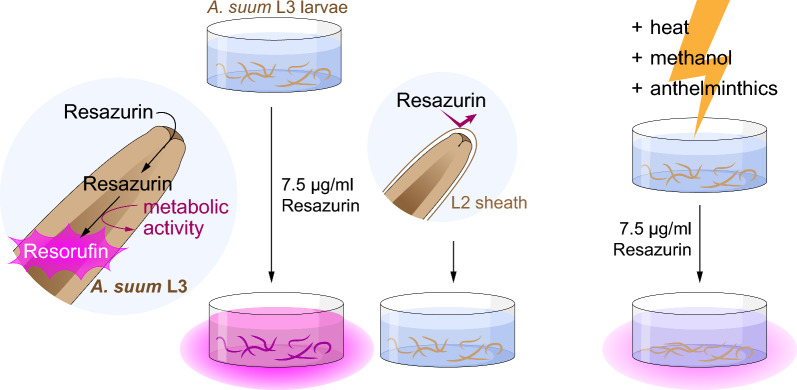

**Supplementary Information:**

The online version contains supplementary material available at 10.1186/s13071-023-05871-5.

## Background

Control of gastrointestinal nematodes primarily relies on chemotherapy in both humans and animals. However, recurrent treatments and reinfections, limitation of currently available anthelmintic drug classes [49], increasing reports of reduced drug efficacy, and evolving resistance for frontline anthelmintics such as the benzimidazoles and ivermectin raise awareness of the importance of anthelmintic drug research [19, 22, 30, 38]. The fast discovery of new drugs against soil-transmitted helminths (STHs) is mainly hindered by the lack of objective high-throughput screening methods for assessing drug effectiveness [1, 20, 47].

Microscopic assessment of motility and morphology dominate the currently used methods for evaluating drug efficacy in larvae. These approaches in general are time-consuming, laborious, and prone to subjectivity, hence not suitable for high-throughput screening. Devices such as the xCELLigence System [40] and the wMicroTracker System™ [24, 37] have been validated for high-throughput drug screening with *Caenorhabditis elegans* and a few parasitic helminth species, yet the readout is limited entirely to locomotion activity. Therefore, combinatorial approaches that address multiple parameters when screening for new drug candidates are discussed, such as a general readout like locomotion activity combined with a specific readout for the putative mode of action, such as electrophysiological or metabolic activity [12].

In addition to motility, nematode viability can be evaluated using indicator dyes of metabolic activity that are converted by larval stages into a measurable product. Different metabolic indicators have been tested for helminths, such as the tetrazolium dye 3-(4,5-dimethyl-2-thiazolyl)-2,5-diphenyltetrazolium bromide (MTT), fluorescein diacetate (FDA), para-nitrophenyl phosphate (pNPP) [34], and the cell-permeable dye resazurin. Resazurin is based on the NADPH-dependent reduction of resazurin to resorufin and widely used to monitor mammalian cell growth and cell metabolism in a wide range of targets [23]. The resazurin reduction assay has been adapted so far for drug screening in adult *Schistosoma* species [25, 26] and in fourth-stage larvae (L4) and adult *Trichuris muris* [39]. To the best of our knowledge, data on the potential of the resazurin reduction assay in *Ascaris* spp. parasites is missing, although it is one of the most common STH worldwide both in human and swine [15, 17, 51].

In this study, we adapt and evaluate the resazurin reduction assay for *Ascaris suum* third-stage larvae (L3). We show that the non-fluorescent resazurin is taken up by viable unsheathed larvae. Resazurin is reduced mainly within the larval midgut to fluorescent resorufin and released into the culture medium. Exposure to commonly used anthelmintics or conditions that compromise the metabolism of *A. suum* L3 were carefully evaluated and resulted in consistent and gradual changes in the resazurin reduction activity. Therefore, the resazurin reduction assay is a promising tool to complement common drug screening methods for detecting effects directly on the metabolic activity of *A. suum* L3.

## Methods

### Collection of *A. suum* eggs

Adult *A. suum* worms were collected bimonthly from a slaughterhouse in Brandenburg (Germany) post-slaughter. Collections were made on days when primarily organic animals were slaughtered. Each batch of worms contained 100–300 worms of different sizes from different pigs. Motile, female worms were separated and washed several times in pre-warmed (37 °C) 0.9% NaCl solution. Worms were transferred into glass bottles at a density of four worms per 200 ml of Hanks' Balanced Salt Solution supplemented with antibiotics (HBSS-AB: 127 mM NaCl, 7.5 mM NaHCO_3_, 5 mM KCl, 1 mM CaCl_2_, 1 mM MgCl_2_ × 6 H_2_O), supplemented with 200 U/ml penicillin, 200 μg/ml streptomycin, 2.5 μg/ml amphotericin B, and 50 μg/ml gentamycin (PAN-Biotech GmbH, Germany), and cultured at 37 °C and 5% CO_2_. After 24 h, worms were transferred into fresh culture medium, and excreted eggs were harvested and collected on a 30-µm cell strainer (Miltenyi Biotec) and washed by flushing the strainer with 50 ml HBSS. Subsequently, eggs were embryonated for 6–8 weeks at 33 °C in 50 ml distilled water (dH_2_O) containing 0.1% formaldehyde and protected from light. The embryonation status was assessed microscopically.

### Flow cytometry of *A. suum* egg development

Each week during embryonation (0–6 weeks), a sample containing 10,000–30,000 *A. suum* eggs was taken for flow cytometric analysis using a FACS Canto II (BD Biosciences) and BD FACSDiva Software v8.0. Without further staining, forward (size) and side scatter (granularity) properties of each sample were recorded and evaluated using FlowJo v10 software (Tree Star).

### Density purification of fully embryonated *A. suum* eggs

Typically, *A. suum* embryonation rates vary between 50 and 95% following 6–8 weeks of embryonation [10, 33] due to some unfertilized eggs and eggs that remain undeveloped in the egg suspension. To overcome this heterogeneity in starting material, fully embryonated *A. suum* eggs were purified using a sucrose density gradient. To reduce egg stickiness and variance in egg density, the uterine layer was removed by washing the eggs three times with dH_2_O (200×*g* at room temperature [RT] for 2 min) and then incubating them in a water bath at 37 °C in 5% HClO (2.4-fold dilution of 12% HClO stock) for 5 min. Subsequently, eggs were washed five times with 50 ml 0.1% bovine serum albumin (BSA) (200×*g* at room temperature for 2 min) and resuspended in 5 ml dH_2_O. A sucrose gradient (18% (w/v), 20%, 23%, 25% each 10 ml) was added using the underlayering step gradient technique. The sucrose gradient was centrifuged at 2000×*g* at room temperature for 1 h in a swing-out rotor (without brake and using soft acceleration). Fully embryonated eggs were recovered from the top of the 23% and 25% sucrose layers using a 20-ml syringe with G20 × 2¾″ needle attached, washed five times with HBSS-AB and pooled for further use.

### Hatching of embryonated *A. suum* eggs

Four millilitres of purified egg suspension was transferred into a 100-ml Erlenmeyer flask containing 9 g of glass beads and placed onto a shaker (100 rpm, 37 °C) for 30 min. This mechanical treatment results in hatching rates of approximately 90%. Hatched *A. suum* L3 were collected on a 30-µm cell strainer, which was then placed in a six-well plate containing HBSS-AB and incubated at 37 °C for 3 h to allow viable larvae to migrate through the sieve. Following migration, larvae were collected, washed twice in HBSS-AB (200×*g* at room temperature for 2 min), and counted.

### Quantifying *A. suum* L3 metabolic activity using resazurin

Resazurin (Acros Organics BV, Belgium) was dissolved in HBSS-AB to prepare a stock concentration of 150 µg/ml. The stock solution was equilibrated at 37 °C and 5% CO_2_ for 24 h, aliquoted, and stored at −20 °C for further use. Fluorescence emission spectra of resazurin and resorufin were measured with a fluorimeter (Cary Eclipse, Agilent).

To titrate the optimal resazurin concentration, 15 µg/ml resazurin stock solution was serially diluted (eight twofold dilution steps), and 10 µl of each dilution was added to each well of a clear, flat-bottom 96-well plate each containing 500 *A. suum* L3 in 90 µl HBSS-AB. The plate was incubated at 37 °C/5% CO_2_ for 24 h, and fluorescence intensity was detected at λ_em_ = 590 nm (Synergy H1, BioTek, λ_ex_ = 540 nm). To quantify the fluorescence signal generated by larval-dependent resazurin reduction, the fluorescence readout was subtracted from a baseline control without *A.* *suum* larvae.

To titrate the optimal larval number, a twofold serial dilution with a range of 1000 to four *A. suum* L3/well was prepared. Resazurin was added to each well at a final concentration of 7.5 µg/ml. The plate was incubated for 24 h, and fluorescence intensity was determined as above.

To address heat-induced changes in metabolic activity, 1.5-ml microcentrifuge tubes with 500 L3 in 500 µl HBSS-AB were placed in a water bath and incubated for varying times (1 to 8 min) at 60 °C and allowed to stand at RT for 20 min before transferring L3 into a 96-well plate with a final volume of 100 µl/well in triplicate. Resazurin was added at a final concentration of 7.5 µg/ml to each well. The plate was incubated at 37 °C/5% CO_2_ for 24 h, and fluorescence intensity was measured as described above. To evaluate methanol-induced changes in larval metabolic activity, 500 L3/well were incubated at 37 °C and 5% CO_2_ for 3 h in HBSS-AB containing 1.5, 3, 6, 12, or 25% (v/v) methanol in triplicate. The plate was incubated for 24 h before determination of fluorescence intensity.

To address the effects of the solvent dimethyl sulfoxide (DMSO) and anthelmintics on larval metabolic activity, DMSO (Sigma-Aldrich, USA) concentrations ranging from 0.5 to 4% (v/v) (final concentrations) were tested. Maximum soluble concentrations of the anthelmintics were assessed using a modified turbidimetric solubility assay [35]. Briefly, stock concentrations of ivermectin (10 mM), mebendazole (10 mM), and thiabendazole (100 mM) in 100% DMSO were serial diluted in DMSO using eight twofold dilution steps. Five microlitres of each dilution step was transferred to 995 µL H_2_O in a 1-ml cuvette, and the turbidity was measured at λ = 600 nm. For anthelmintic treatments, ivermectin (Sigma-Aldrich, USA) at 6.25 µM and mebendazole (Sigma-Aldrich, USA) at 50 µM were dissolved in 5% (v/v) DMSO in HBSS-AB to prepare 10-fold stock solutions. Thiabendazole (Sigma-Aldrich Corp., USA) was dissolved at 1 mM, 0.1 mM, and 0.01 mM in 5% DMSO in HBSS-AB. Ten microlitres of each DMSO dilution or drug stock solution was added to wells of a clear, flat-bottom 96-well plate each containing 500 *A. suum* L3 in 90 µl HBSS-AB. The plates were incubated for 24 h at 37 °C and 5% CO_2_. Thereafter, 5.3 µl resazurin (final conc. 7.5 µg/ml) was added to each well, and plates were incubated at 37 °C and 5% CO_2_ for 24 h. The fluorescence intensity was detected as described above.

### Microscopic evaluation of resazurin reduction site

To assess the site of resazurin reduction within the larvae, *A. suum* L3 were incubated in HBSS-AB containing 7.5 µg/ml resazurin at 37 °C and 5% CO_2_ for 3 h. Larvae were washed three times with ice-cold dH_2_O and imaged with a fluorescence microscope (Axio Vert.A1 with Colibri 7, Zeiss) using an excitation wavelength of λ_ex_ = 555 nm and a band-pass filter of λ_em_ = 579-604 nm. To verify pharyngeal pumping in hatched *A. suum* L3, larvae were incubated in HBSS-AB containing 10 mg/ml fluorescein-conjugated bovine serum albumin (FITC-BSA; Thermo Fisher Scientific Inc., USA) for 3 h and subsequently washed three times in ice-cold dH_2_O [7]. Fluorescence was detected using an excitation wavelength of λ_ex_ = 475 nm and a bandpass filter of λ_em_ = 500–525 nm (Axio Vert.A1 with Colibri 7, Zeiss).

### Statistical analysis

GraphPad Prism 9 was used for regression analysis and the comparison of groups. A linear regression model was used for the data obtained from the L3 titration experiment, and a hyperbolic regression model was used for the data obtained from the resazurin titration experiment. Regression parameters were estimated using the method of least squares. Regression models were considered adequate to the data when *R*^2^ was above 0.8. A paired Student *t*-test was used to analyse the statistical significance of the amount of resorufin released into the media from the larvae. After a simple one-way analysis of variance (ANOVA), Dunnett’s test was used to compare fluorescence intensity data obtained from the experiments in which the impact of heat, methanol, DMSO, and thiabendazole on L3 was measured. The statistical impact of ivermectin and mebendazole was analysed using an unpaired *t*-test.

## Results

### Density purification of fully embryonated *A. suum* eggs

**Fig. 1:**
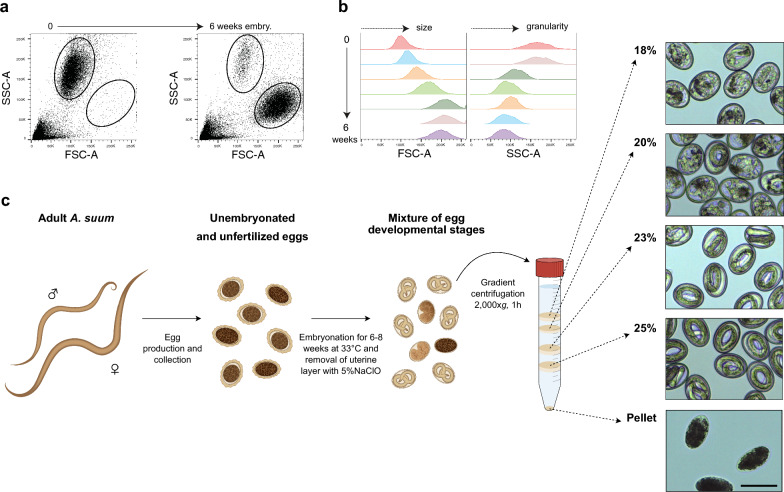
Purification of embryonated *A.* *suum* eggs using sucrose density gradient centrifugation. **a** Exemplary flow cytometric scatter plots depicting forward (FSC-A) and sideward scatter (SSC-A) properties of un-embryonated *A.* *suum* eggs in suspension before embryonation (left) and a mixed egg suspension after 6 weeks of embryonation (right). **b** Histograms of weekly FSC/SSC measurements of *A.* *suum* eggs from week 0 to week 6 after start of embryonation. **c** Workflow for separating fully embryonated *A.* *suum* eggs from undeveloped eggs. The uterine layer is removed prior to gradient purification. Mixed egg suspensions are separated using four sucrose layers—18% (w/v), 20%, 23%, and 25%—and eggs are evaluated microscopically (black scale bar: 50 µm) for each layer

Continuous embryogenesis of *A.* *suum* eggs results in a heterogenous mixture of developmental stages such as unfertilized eggs, 1–8-cell-stage eggs, or fully embryonated eggs in a given batch. Using flow cytometry, we analysed the egg mixture over the course of 6 weeks during embryonation. Analysing forward- and side-scatter properties of these eggs derived from gravid female uteri revealed gradual changes in egg morphology, namely an increase in egg size with a concomitant decrease in granularity (Fig. 1a, b). Hypothesizing that egg granularity affects egg density, we used a discontinuous, multi-layered sucrose density gradient [25% (w/v), 23%, 20%, 18%] in order to separate the different embryonation stages of the eggs. The sucrose gradient reproducibly fractionated egg mixtures into four distinct layers and a pellet. Microscopic evaluation (Fig. 1c) revealed that eggs collected from the top of the two upper layers (18% and 20% sucrose) contained mostly pre-larval stages, while the pellet consisted mostly of unfertilized eggs and some already hatched larvae. In turn, both the 23% and 25% sucrose layers harboured eggs with fully developed larvae inside and with a high purity of > 95%. Of note, larvae inside eggs that we obtained from both the 23% and 25% sucrose layers appeared fully embryonated and were therefore pooled and used for all subsequent in vitro assays.

### Viable *A. suum* L3 reduce resazurin to resorufin

**Fig. 2:**
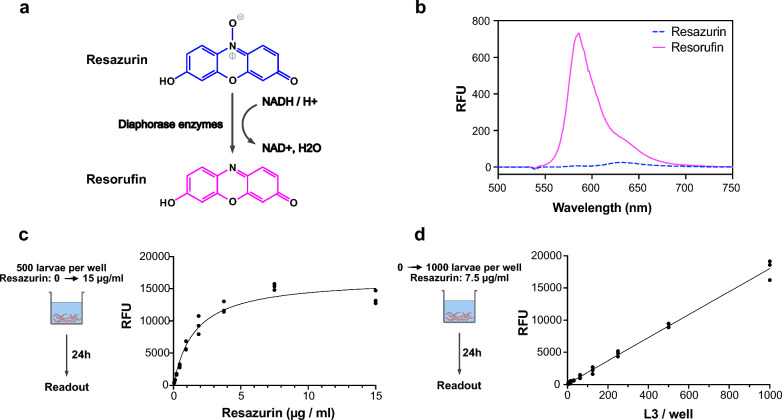
*Ascaris suum* L3 reduce the metabolic indicator dye resazurin. **a** Biochemical reaction in which the non-fluorescent dye resazurin (blue) is reduced to fluorescent resorufin (pink). The reaction requires the presence of diaphorase enzymes and NADH/H+. **b** Fluorescence emission spectra of resazurin (blue dashed curve) and resorufin (pink solid curve) in HBSS-AB at λex = 540 nm. **c** Titration of resazurin from 0 to 15 µg/ml with a fixed number of 500 in vitro-hatched *A.* *suum* larvae per well shows a hyperbolic relationship (*df* = 7, *R*^2^ = 0.98, best-fit ½max = 1.6 µg/ml). **d** Titration of *A.* *suum* L3 numbers from 0 to 1000 in vitro-hatched L3 with the fixed concentration of 7.5 µg/ml resazurin reveals a strong linear correlation (*R*^2^ = 0.99, equation: *Y* = 17.89x + 122.3, *r* = 0.99, with *P* < 0.0001). **c**, **d** Fluorescence intensity measured at λem = 590 nm and λex = 540 nm. Black dots represent fluorescence intensity from single wells (*n* = 3 technical replicates). Grey curve represents the curve of best fit (least-squares method)

Since cell viability can be measured by fluorometry as a result of the diaphorase enzyme-dependent reduction of resazurin to resorufin (Fig. 2a), we aimed to analyse if this enzymatic reaction ca be applied to detect *A. suum* larval metabolic activity. The excitation wavelength for resorufin is within the range of 530–570 nm. For our experiments, we set the excitation wavelength to 540 nm and measured the fluorescence emission spectra of resorufin at λex=540 nm to detect the maximum fluorescence signal intensity, which was at 590 nm (Fig. 2b).

Titration assays were performed to determine optimal ranges of both the concentration of resazurin and the number of *A. suum* larvae per assay. Resazurin concentration was titrated from 15 to 0.06 µg/ml, with a constant number of 500 larvae per well (Fig. 2c). Using the least-squares fit method, a hyperbolic regression model provided the best fit with *R*^2^ = 0.98 (*df* = 7, RFU = 16,657, 95% CI: RFU = 14,574–19,104). Based on the hyperbolic regression curve obtained, the half-maximal resazurin concentration was at 1.6 µg/ml (95% CI: ½max = 1.038–2.44 µg/ml). However, a resazurin concentration of 7.5 µg/ml provided a better signal resolution with a 1.83-fold stronger signal intensity without visibly affecting larval motility at the same time. To titrate optimal larval numbers, we performed a serial dilution of 1000 to four *A. suum* L3 with a constant resazurin concentration of 7.5 µg/ml (Fig. 2d). Using the least-squares fit method, a simple linear regression model provided the best fit, with *Y* = 17.89x + 122.3. In addition, Pearson correlation analysis confirmed a strong linear relationship between number of larvae and fluorescence intensity with *r* = 0.99 and *P* < 0.0001. We determined 100 to 1000 to be optimal numbers of *A.* *suum* L3 used in a 96-well assay.

**Fig. 3:**
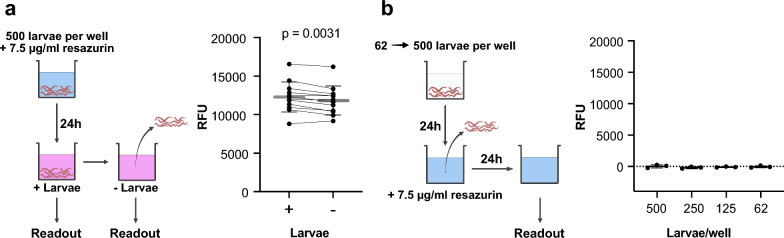
*Ascaris suum* L3 reduce resazurin to resorufin and release the majority into the media. **a** Comparison of fluorescence intensity before and after larval removal: 500 *A.* *suum* L3 per well were measured after incubation with 7.5 µg/ml resazurin for 24 h (+ larvae) and after removal of L3 (− larvae). Both groups (*n* = 12 technical replicates) were compared using the paired *t*-test (*t* = 3.774, *df* = 11, *P* = 0.0031). **b** Excretory–secretory (ES) products of *A.* *suum* L3 do not convert resazurin into resorufin. Fluorescence intensity of ES-containing supernatants collected from 62, 125, 250, and 500 L3/well after incubation with 7.5 µg/ml resazurin for 24 h. Scatter plot shows mean and standard deviation (SD) (solid grey line) of relative fluorescence units (RFUs), black dots represent RFUs of single wells, and the dotted line represents *y* = 0; *n* = 3 technical replicates

To assess whether the fluorescent product resorufin accumulates in the larvae or is released into the medium, we measured fluorescence intensity after 24 h with *A.* *suum* L3 remaining in the wells and compared that to supernatants from which L3 had been removed (Fig. 3a). Although our data revealed a decrease in fluorescence intensity between both measurements (paired *t*-test: *n* = 12, *t* = 3.774, *df* = 7, *P* = 0.0031), the mean fluorescence intensity after larval removal was reduced by 3.8% compared to the mean fluorescence intensity of medium samples with larvae being present during the measurement. Thus, the vast majority of resorufin was detected in the medium. Furthermore, supernatants collected after incubation of 62, 125, 250, and 500 L3/96-well for 24 h did not show detectable potential for reduction of resazurin to resorufin (Fig. 3b). The latter finding indicated that resazurin is reduced inside the larvae.

### Localization of resazurin reduction inside *A. suum* L3

**Fig. 4:**
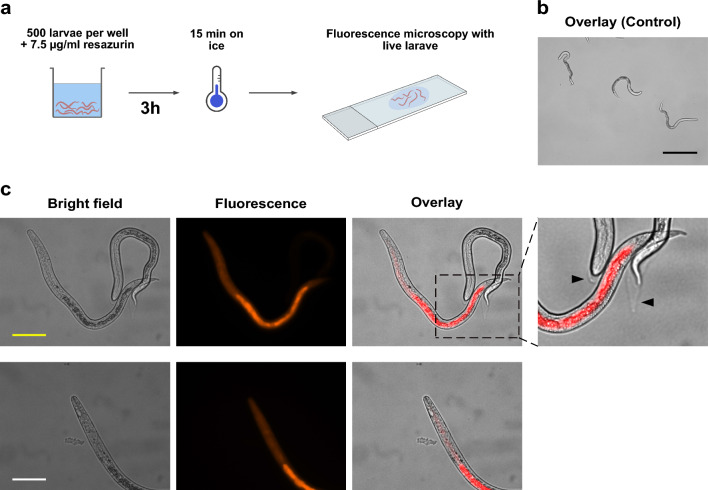
Resorufin localization inside *A. suum* L3. **a** Experimental set-up for localization of resorufin inside living larvae by fluorescence microscopy. **b** Control larvae incubated without resazurin. **c** Image acquisition 3 h after addition of 7.5 µg/ml resazurin. Bright field (left), fluorescence (middle), and overlay (right) images of resorufin inside unsheathed L3. A magnified view of the posterior and anterior end of a non-fluorescent larva depicting the L2 cuticle around the L3 (black arrow heads). Image acquisition settings: λex = 555 nm with a bandpass filter for λem = 579–604 nm. Black scale bar: 100 µm, yellow scale bar: 40 µm, white scale bar: 20 µm

Next, we imaged *A. suum* L3 incubated with resazurin for 3 h using a fluorescence microscope (Fig. 4a) to visualize the tissue where resazurin reduction in the larvae occurs. Within the *A. suum* L3, we observed a bright fluorescence signal which was unevenly distributed inside the larval body. A coherent bright signal was emitted from a region inside the larvae at the posterior end, which we, based on morphological descriptions of Kirchgäßner et al. [21], allocate to the worms’ midgut. Larvae incubated without resazurin showed no autofluorescence at λ_ex_ = 555 nm (Fig. 4b).

Some larvae did not show any fluorescence signals, although they appeared vital and motile throughout the assay (Fig. 4c). Further analysis of the non-fluorescent L3 revealed a transparent, loose sheath visible at both the anterior and posterior larval ends (Fig. 4c, black arrowheads). We therefore conclude that the sheath that Geenen et al. [11] referred to as cuticula of the L2 and that occasionally remains after the second molt as well as after mechanical hatching prevents the uptake of resazurin by viable *A.* *suum* L3.

Pharyngeal pumping of hatched *A. suum* L3 was verified by incubating the larvae with FITC-BSA. Fluorescence was observed from the anterior end and mainly from the gut in unsheathed larvae, indicating a gut-focused localization. Ensheathed larvae did not exhibit fluorescence (Additional file 1: Fig. S1).

### Impairment of *A. suum* L3 metabolic activity by heat treatment and methanol

**Fig. 5:**
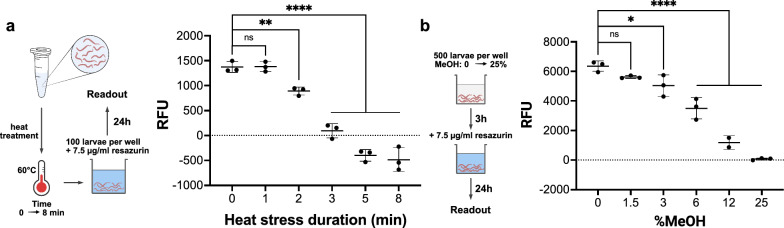
Resazurin reduction assay enables quantification of metabolic impairment. Metabolic activity was assessed for *A. suum* larvae after **a** heat treatment at 60 °C for 0 to 8 min and **b** exposure to different methanol concentrations for 3 h using the resazurin reduction assay. Scatter plots represent mean (SD) fluorescence measurements of *n* = 3 technical replicates per treatment. Asterisks indicate statistical significance by Dunnett’s test: *P < 0.05, **P < 0.01, ****P < 0.0001; ns: not statistically significant

Naidoo et al. [27] have shown that temporary exposure of viable *A. suum* eggs to 60 °C for various times results in a gradual decrease in egg viability, which was assessed by morphology and motility. Furthermore, methanol-induced feeding inhibition has been reported with gradual effects at concentrations from 1 to 5% [18, 42] and a lethal impact for methanol concentrations from 50% in *C. elegans* [8]. Therefore, we exposed *A.* *suum* L3 to heat for variable times (Fig. 5a) and methanol at variable concentrations (Fig. 5b) to test the ability of the resazurin reduction assay to detect varying degrees of damage of the larvae. We observed that 60 °C treatment for longer than 3 min significantly reduced the metabolic activity of *A.* *suum* L3 by more than 93% (Dunnett’s test, *df* = 12, 3 min: *q* = 11.16, 5 min: *q* = 15.48, 8 min: *q* = 16.22, *P* ≤ 0.0001). Shorter treatment times (2 min) led to a reduction of 50% (Dunnett’s test, *df* = 12, *q* = 4.226, *P* ≤ 0.01), while 1 min of 60 °C treatment did not induce changes in the metabolic activity of *A.* *suum* L3 (Dunnett’s test, *df* = 12, *q* = 0.055, *P* > 0.99). For larvae treated with 1.5 vol% methanol for 24 h, no significant impact on resazurin reduction activity was detected. However, gradual impairment of resazurin reduction activity was observed at concentrations of 3%, 6%, and 12%, resulting in significantly weaker fluorescence signals by 20% (*P* ≤ 0.05), 45% (*P* ≤ 0.0001), and 70% (*P* ≤ 0.0001), respectively. Methanol at 25% completely abrogated resazurin reduction. In the control, methanol itself at concentrations of 25% did not notably change fluorescence characteristics of resorufin (Additional file 2: Fig. S2). A gradual decrease in resorufin production was measured with increasing methanol concentrations. Four-parameter logistic regression revealed a half-maximal effective concentration (EC_50_) = 6.8% (*df* = 26, *R*^2^ = 0.95, 95% CI for EC_50_ = 4.3–9.2%) for methanol (Additional file 3: Fig. S3). Overall, for *A. suum* L3 exposed for increasing times to 60 °C or increasing methanol concentrations, we observed a gradually measurable decrease in resazurin reduction activity in the larvae.

### Impact of anthelmintic drugs on the metabolism of *A. suum* L3

We further evaluated the resazurin reduction assay regarding its ability to determine effects of anthelmintic drug exposure in vitro on hatched *A. suum* L3. Since DMSO was used as drug solvent and has been reported to effect mammalian cell viability already at low concentrations at 1–8 vol% DMSO [9], we first assessed the effects of DMSO on *A. suum* L3 metabolism.

For DMSO concentrations of 0.5% and 1%, larval metabolism was not significantly affected when comparing mean fluorescence intensities of treated against untreated larvae using the Dunnett’s multiple-comparisons test. However, mean fluorescence intensity was reduced by 19.5% for 0.5% (*df* = 10, *q* = 2.328, *P* = 0.124) and by 17.2% for 1% (*df* = 10, *q* = 2.05, *P* = 0.1907) of DMSO. A DMSO concentration of 2% resulted in a significant decrease by 26% (*df* = 10, *q* = 3.163, *P* = 0.032) of larval metabolic activity, while exposures up to 4% DMSO resulted in immobilization of the larvae and 58% decrease (*df* = 10, *q* = 6.971, *P* = 0.0002) of resazurin reduction activity. To reduce the impact of DMSO on larval metabolism, we considered a DMSO concentration of 0.5% for the following anthelmintic drug testing.

A significant reduction in larval metabolic activity was observed for all three anthelmintics at different concentrations. Exposure of the larvae to 1 µM thiabendazole resulted in a non-significant decrease in resazurin reduction activity by 22.7% (Dunnett’s test, *df* = 8, *q* = 2.207, *P* = 0.1354). Thiabendazole at concentrations of 10 µM and 100 µM significantly reduced larval resazurin reduction activity by 70% (Dunnett’s test, *df* = 8, *q* = 6.771, *P* = 0.0004) and 89% (Dunnett’s test, *df* = 8, *q* = 8.647, *P* < 0.0001) compared to the untreated group, respectively. Mebendazole at 5 µM and ivermectin at 0.625 µM significantly reduced larval reduction activity by 73% (*t*-test, *t* = 9, *df* = 4, *P* < 0.0006) and 55% (*t*-test, *t* = 11.66, *df* = 4, *P* < 0.003), respectively.

## Discussion

In the present study, we evaluated the potential of the resazurin reduction assay for measuring metabolic activity in *A.* *suum* L3. Since intracellular resazurin reduction depends on the activity of diaphorase enzymes [32], it has been widely used as an indicator in viability, proliferation, and toxicity assays [2, 29, 31, 52]. A few studies have reported on the attempts to use the redox indicator resazurin for drug screening in helminths, including *Schistosoma* species [25, 26], *T. muris* [39], and *Ancylostoma ceylanicum* [43]. However, to the best of our knowledge, data evaluating the resazurin reduction assay for *A.* spp. L3 is missing, although it belongs, like *Trichuris* spp., to the most prevalent STH worldwide affecting humans and animals alike [15, 51].

Our results show that resazurin is reduced inside *A.* *suum* larvae to the fluorescent resorufin. Resorufin production is directly proportional to the number of *A.* *suum* L3, which also has been described for schistosomula [26]. We observed that hyperbolic saturation of fluorescence intensity occurs with increasing resazurin concentration, which is indicative of an enzyme-mediated reduction of resazurin, by intracellular diaphorase enzymes [32, 48]. Resazurin-induced toxicity at 7.5 µg/ml can probably be excluded since concentrations of up to 12.5 µg/ml were reported to be non-toxic for mammalian cells [23]. However, we observed reduced motility in *A.* *suum* L3 at resazurin concentrations above 15 µg/ml, which needs further investigation.

Further, we aimed to localize the site at which resazurin is reduced to resorufin by the parasite. We considered the possibility of resazurin reduction by either excretory/secretory (ES) products or the content of extracellular vesicles. ES-containing supernatant from *A.* *suum* L3 cultures incubated with resazurin showed no concentration-dependent increase in fluorescence intensity, controlling for the possibility of resazurin reduction by ES products or extracellular vesicle contents that were reported to contain metabolic enzymes [13, 45]. Based on the observations of FITC-BSA uptake restriction in ensheathed *A. suum* L3 (Additional file 1: Fig. S1) and spatial overlap of the fluorescence signals from L3 incubated with FITC-BSA or resazurin, we assume that resazurin is taken up through the oral opening of unsheathed larvae rather than through the cuticula and epidermis. Furthermore, the presence of ensheathed larvae could bias the evaluation of drug screening assays, especially when focusing on motility readouts only, since the cuticle can affect drug uptake. Based on this observation and similar reports on L3 of *Ancylostoma caninum* and *Heligmosomoides polygyrus* [6, 7], we suggest to carefully examine frequency of unsheathed larvae after hatching to reduce a possible bias for *A. suum* L3 in vitro assays. Alternatively, it might be possible to normalize data to the mean of the vehicle controls obtained within the same batch of larvae before comparing between different batches.

**Fig. 6:**
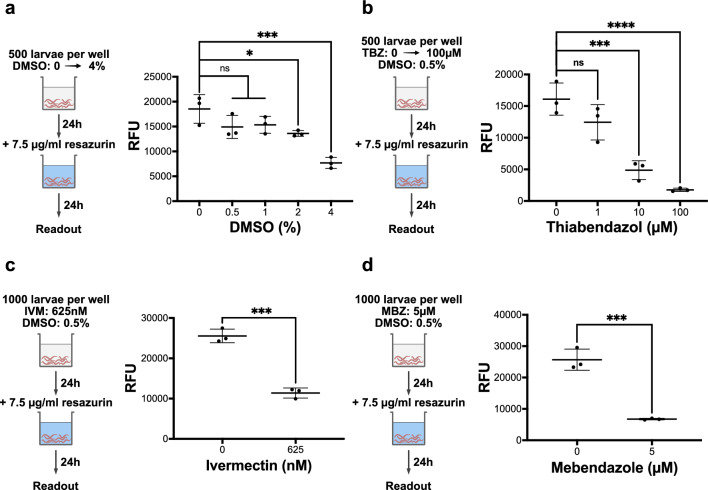
Resazurin reduction assay in drug testing. Larvae were exposed to anthelmintics for 24 h, following an incubation with 7.5 µg/ml resazurin for 24 h. The relative fluorescence intensities were measured fluorometrically for **a** DMSO-treated larvae from 0 to 4 vol%. **b** Thiabendazole in 0.5% DMSO applied from 0 to 100 µM. **c** Ivermectin in 0.5% DMSO applied at 625 nM. **d** Mebendazole in 0.5% DMSO applied at 5 µM. Scatter plots represent the mean fluorescence intensity (λex = 540, λem = 590) for *n* = 3 technical replicates. Asterisks indicate statistical significance by Dunnett’s test for DMSO and thiabendazole and by t-test for ivermectin and mebendazole: *P ≤ 0.05, ***P ≤ 0.001, ****P ≤ 0.0001; ns: not statistically significant. All drug assays were independently repeated using in vitro-hatched *A. suum* L3 from different batches

Heat-treated *A. suum* L3 in our study gradually produced less resorufin with increasing heat exposure time, presumably due to heat-induced impairment of larval metabolic activity. We observed complete inactivation of metabolism in larvae treated longer than 3 min. Similar results were reported by Naidoo et al. [27, 28], who observed inactivation of egg viability and visible egg damage after 3 min of exposure to 60 °C. Further, we evaluated the impact of methanol on *A. suum* L3 for concentrations up to 25 vol% using the resazurin reduction assay. In line with observations of Jones and Candido [18], our results show gradually increasing toxic effects on larval metabolic activity at methanol concentrations from 3 to 12% and an almost complete metabolic inactivation at 25%. However, our findings show that *A. suum* L3 are more susceptible to methanol than *C. elegans*, since the reported 24-h LC_50_ value of 12% for *C. elegans* is twice as high as for *A. suum* L3 with 6.8% methanol (Additional file 3: Fig. S3). In conclusion, the resazurin reduction assay can be used to measure the metabolic activity of *A. suum* L3 and help in detecting subtle changes of metabolism when physical or chemical stress is applied. However, since the assay measures the overall reduction potential of 100 or more larvae, it still needs to be investigated whether the observed fluorescence decrease occurs due to a subpopulation of dead larvae or a metabolic impairment of the whole initial larval population after the applied stress. In addition, it should be ensured that the agent to be tested does not have any strong reducing properties and does not change the pH value of the medium used to avoid oversaturation of the fluorescence signal and/or erroneous measurements (Fig. 6).

Further, we evaluated the resazurin reduction assay for its ability of assessing anthelmintic drug effects in *A. suum* L3. We tested ivermectin, mebendazole, and thiabendazole, prominent examples of the macrocyclic lactone and benzimidazole drug classes primarily used for anthelmintic chemotherapy, and effective against ascariasis in humans and pigs [4, 5, 41, 46]. Since these anthelmintics have very poor solubility properties in aqueous solutions, DMSO is mostly added to increase solubility. However, toxic effects of DMSO have been reported for 2% to 4% for mammalian cells [9]. Hence, we investigated the impact of DMSO for volume concentrations up to 4% and found that the limit of DMSO concentrations not significantly affecting the larval metabolism is 0.5–1%. The in vitro effect of ivermectin on *A. suum* larvae was evaluated previously using motility and migration assays [14, 16]. Hu et al. [16] reported for ivermectin a high IC_50_ value of > 1.14 mM in *A.* *suum* L4 (intestinal isolates 14 days post-infection). However, we were not able to reproduce an ivermectin concentration of 1.14 mM without precipitation of the drug in 0.5% DMSO. Therefore, we titrated ivermectin in 0.5% DMSO (in H_2_O) and detected turbidimetrically its maximum solubility at 1.56 µM in H_2_O (Additional file 4: Table S1). Moreover, the solubility of ivermectin in HBSS-AB was lower due to medium components like salts, sugar and antibiotics, which reduced the maximum soluble concentration to 625 nM. At this concentration of ivermectin, we observed 55% reduction in the activity of resazurin reduction in the L3, which consequently indicates a considerably lower EC_50_ value for *A. suum* L3 than for *A.* *suum* L4 observed by Hu et al. [16]. Hansen et al. [14] reported for ivermectin an IC_50_ of ~ 1 µM in *A.* *suum* L3, which is closer to our observations. However, for *C. elegans*, significant effects were observed at considerably lower ivermectin concentrations (0.6–20 nM ivermectin) [3, 12]. On the one hand, this may indicate that the resazurin reduction assay is less sensitive for detecting the effects of ivermectin on *A. suum* L3 compared to in vitro assays in other nematodes. On the other hand, together with the observations from Hu et al. [16] and Hansen et al. [14], it is conceivable that *A. suum* larvae are less susceptible to ivermectin than *C. elegans* larvae. The impact of thiabendazole and mebendazole on *A. suum* L3 have been investigated so far by Zhao et al. [50], who reported an EC_50_ values in the range of 2.3–150 nM using an agar migration assay for drug effect evaluation after 24 h drug treatment. However, 1000-fold higher concentrations of these anthelmintics resulted in significant but not complete inactivation of resazurin reduction activity by 73% to 89% in the present study. The direct comparison of the studies is difficult because the drug effects were evaluated using different methods. However, the resazurin reduction assay might be less sensitive in detecting impairments of larval locomotion rather than the agar migration assay but might provide a better sensitivity for detecting impairments of larval metabolic activity.

The embryonation rates of *A. suum* eggs can vary from batch to batch, which results in a heterogeneous egg solution, that comprises infertile, unembryonated, and fully embryonated eggs [10, 33]. If partially developed larvae remain in the final population, this could bias drug screening results of motility, migration, or metabolic activity assays. Therefore, synchronization of a particular larval development stage is one solution for minimizing the variability of any assay involving nematode larvae, as described for the free-living nematode *C. elegans* [36]. However, to the best of our knowledge, such approaches in *A. suum* L3 have not been investigated. Using a discontinuous, multi-layered sucrose density gradient, we separated and enriched eggs of different development stages from a heterogenous egg solution. Interestingly, fully embryonated eggs obtained from the layers of 23% and 25% sucrose showed similar and typical morphology of L3 [44]. Further investigations are required to examine possible differences between those two density types of eggs.

## Conclusion

In conclusion, this study established the resazurin reduction assay for quantification of the metabolic activity in *A. suum* L3 and demonstrates for the first time a straightforward approach for enrichment of fully embryonated *A. suum* eggs. The resazurin reduction assay can detect gradual metabolic changes of *A. suum* L3 after exposure to physical and chemical stressors, including major anthelmintic drugs. The assay is inexpensive and simple and, when used correctly, can drive medium-throughput drug screening. Additionally, it may provide insights on general larval metabolic activity. The resazurin reduction assay has an excellent potential to complement available assays for screening for drug effects and has particularly the advantage to reduce a potential readout bias and advance more simple combinatorial screening approaches for putative anthelmintics, which ultimately can accelerate anthelmintic drug discovery.

## Supplementary Information

**Additional file 1: Figure S1. **Unsheathed *A. suum* L3 ingest fluorescein-conjugated bovine serum albumin (FITC-BSA). Strong fluorescence is exhibited from **a** the midgut (left) and the oral opening (right). **b** L3 carrying an intact sheath (left, indicated by black arrowhead) do not exhibit fluorescence (right). Image acquisition settings: λ_ex_ = 475 nm with a bandpass filter for λ_em_ = 500–525 nm. White scale bar: 20 µm. Yellow scale bar: 40 µm.

**Additional file 2: Figure S2. **Fluorescence emission spectra of resorufin diluted in H_2_O and 25% methanol (MeOH) at 540 nm. The dashed black curve (peak at 585 nm) represents the measured emission spectrum of resorufin diluted in H_2_O, and the solid black curve (peak at 587 nm) represents the measured emission spectra of resorufin diluted in 25% MeOH.

**Additional file 3: Figure S3. **Impact of methanol (MeOH) on metabolic activity of *A. suum* L3. Larvae (500 L3/96-well) were exposed for 3 h to different concentrations (v/v) of MeOH, and the relative fluorescence intensity of resorufin was measured after incubation with 7.5 µg/ml resazurin for 24 h. Four-parameter logistic regression analysis on log10-transformed MeOH concentration was used to interpolate dose response curve (solid black line; *df* = 26, *R*^2^ = 0.95). Best-fit EC_50_ = 6.8% (95% CI = 4.3–9.2%). Black dots represent arithmetic means, and whiskers correspond to the standard deviation of *n* = 3 technical replicates. Coordinates plotted using base-2 logarithmic scale on the *x*-axis and linear scale on the *y*-axis.

**Additional file 4: Table S1.** Maximum soluble concentrations of ivermectin, mebendazole, and thiabendazole in 0.5% DMSO determined by turbidimetric solubility assay.

## Data Availability

All data supporting the conclusions of this article are included within the article and its additional files.

## Abbreviations

BSA: Bovine serum albumin
dH_2_O: Distilled water
ES: Excretory/secretory
FDA: Fluorescein diacetate
FITC-BSA: Fluorescein-conjugated bovine serum albumin
HBSS-AB: Hank's Balanced Salt Solution supplemented with antibiotics
L3: Third-stage larvae
L4: Fourth-stage larvae
MTT: 3-(4,5-Dimethyl-2-thiazolyl)-2,5-diphenyltetrazoliumbromid
pNPP: para-Nitrophenyl phosphate
STH: Soil-transmitted helminth
v/v: Volume concentration
w/v: Mass concentration
