# Supplementary figures and images for: Quantifying metabolic activity of Ascaris suum L3 using resazurin reduction
Source: Parasit Vectors. 2023 Jul 19;16:243. doi: 10.1186/s13071-023-05871-5 (PMC10357624; doi:10.1186/s13071-023-05871-5)

**a**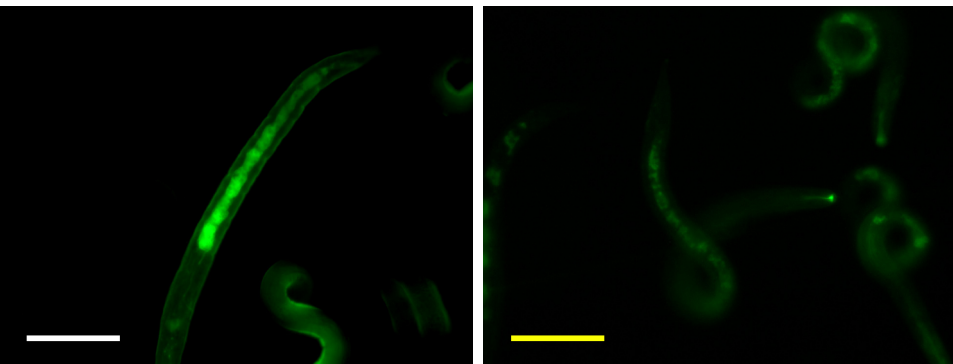**b**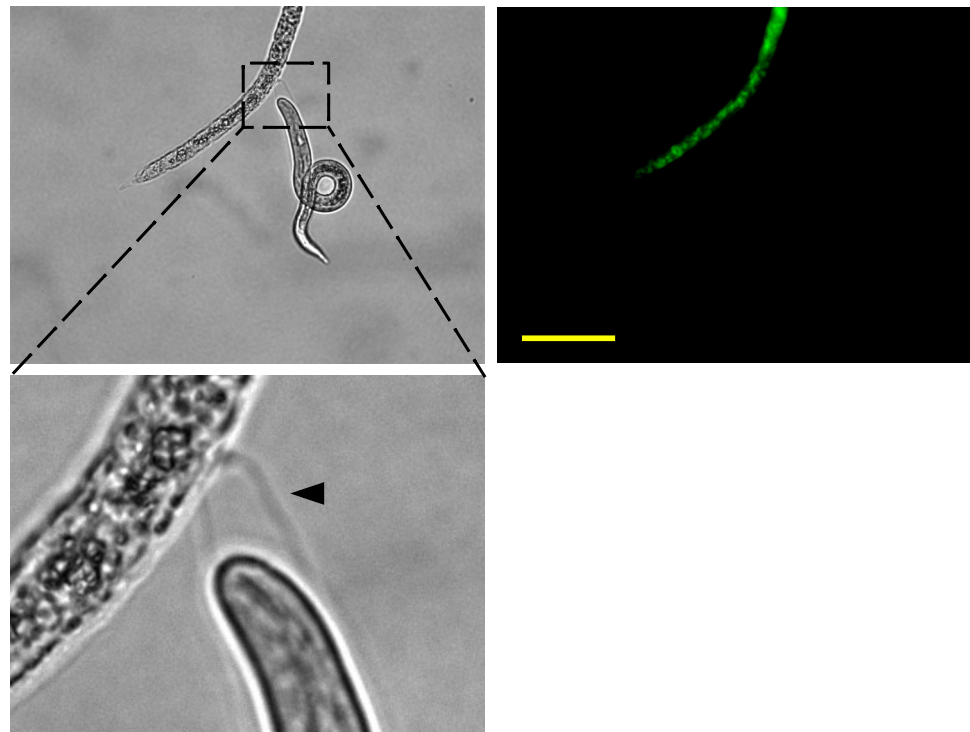

Supplement: Supplementary file 1 — Additional file 1: Figure S1. Unsheathed A. suum L3 ingest fluorescein-conjugated bovine serum albumin (FITC-BSA). Strong fluorescence is exhibited from a the midgut (left) and the oral opening (right). b L3 carrying an intact sheath (left, indicated by black arrowhead) do not exhibit fluorescence (right). Image acquisition settings: λex = 475 nm with a bandpass filter for λem = 500–525 nm. White scale bar: 20 µm. Yellow scale bar: 40 µm. [file 13071_2023_5871_MOESM1_ESM.pdf]

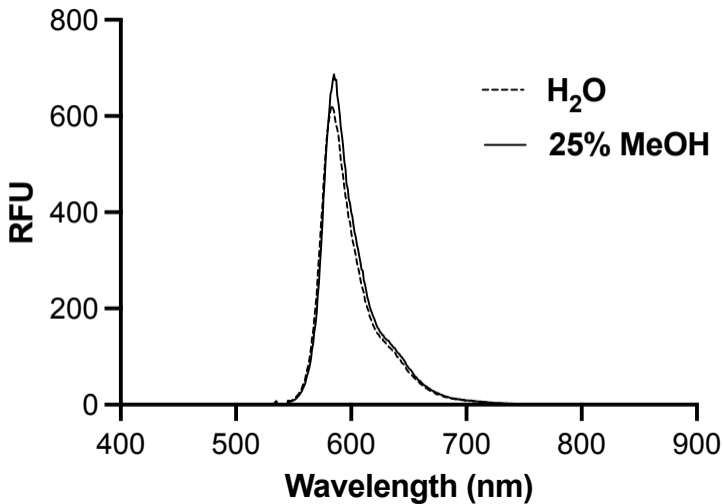

Supplement: Supplementary file 2 — Additional file 2: Figure S2. Fluorescence emission spectra of resorufin diluted in H2O and 25% methanol (MeOH) at 540 nm. The dashed black curve (peak at 585 nm) represents the measured emission spectrum of resorufin diluted in H2O, and the solid black curve (peak at 587 nm) represents the measured emission spectra of resorufin diluted in 25% MeOH. [file 13071_2023_5871_MOESM2_ESM.pdf]

RFU

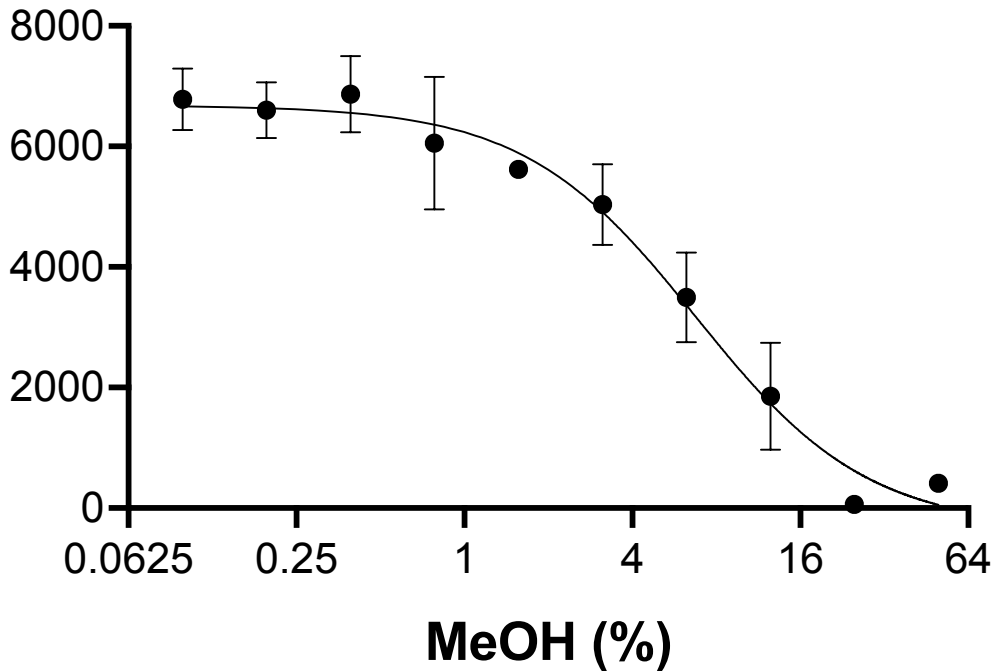

Supplement: Supplementary file 3 — Additional file 3: Figure S3. Impact of methanol (MeOH) on metabolic activity of A. suum L3. Larvae (500 L3/96-well) were exposed for 3 h to different concentrations (v/v) of MeOH, and the relative fluorescence intensity of resorufin was measured after incubation with 7.5 µg/ml resazurin for 24 h. Four-parameter logistic regression analysis on log10-transformed MeOH concentration was used to interpolate dose response curve (solid black line; df = 26, R2 = 0.95). Best-fit EC50 = 6.8% (95% CI = 4.3–9.2%). Black dots represent arithmetic means, and whiskers correspond to the standard deviation of n = 3 technical replicates. Coordinates plotted using base-2 logarithmic scale on the x-axis and linear scale on the y-axis. [file 13071_2023_5871_MOESM3_ESM.pdf]
