# Supplementary material for: Quantifying metabolic activity of Ascaris suum L3 using resazurin reduction
Source: Parasit Vectors. 2023 Jul 19;16:243. doi: 10.1186/s13071-023-05871-5 (PMC10357624; doi:10.1186/s13071-023-05871-5)
Supplement: Supplementary file 4 — Additional file 4: Table S1. Maximum soluble concentrations of ivermectin, mebendazole, and thiabendazole in 0.5% DMSO determined by turbidimetric solubility assay. [file 13071_2023_5871_MOESM4_ESM.docx]

| **Anthelmintic** | **Maximum soluble concentration in 0.5% DMSO (µM)** |
| --- | --- |
| Ivermectin | 1.56 |
| Mebendazole | 6.2 |
| Thiabendazole | 100 |
